# Untargeted urine metabolomics and machine learning provide potential metabolic signatures in children with autism spectrum disorder

**DOI:** 10.3389/fpsyt.2024.1261617

**Published:** 2024-02-20

**Authors:** Xian Liu, Xin Sun, Cheng Guo, Zhi-Fang Huang, Yi-Ru Chen, Fang-Mei Feng, Li-Jie Wu, Wen-Xiong Chen

**Affiliations:** ^1^ Department of Children’s and Adolescent Health, College of Public Health, Harbin Medical University, Harbin, China; ^2^ Division of Birth Cohort Study, Guangzhou Women and Children’s Medical Center, Guangzhou Medical University, Guangdong Provincial Clinical Research Center for Child Health, Guangzhou, China; ^3^ Clinical Research and Innovation Center, Xinhua Hospital Affiliated with Shanghai Jiao Tong University, Shanghai, China; ^4^ The Assessment and Intervention Center for Autistic Children, Guangzhou Women and Children’s Medical Center, Guangzhou Women and Children’s Medical Center, Guangzhou Medical University, Guangdong Provincial Clinical Research Center for Child Health, Guangzhou, China; ^5^ Department of Neurology, Guangzhou Women and Children’s Medical Center, Guangzhou Medical University, Guangdong Provincial Clinical Research Center for Child Health, Guangzhou, China

**Keywords:** autism, untargeted metabolomics, machine learning, urine, screen

## Abstract

**Background:**

Complementary to traditional biostatistics, the integration of untargeted urine metabolomic profiling with Machine Learning (ML) has the potential to unveil metabolic profiles crucial for understanding diseases. However, the application of this approach in autism remains underexplored. Our objective was to delve into the metabolic profiles of autism utilizing a comprehensive untargeted metabolomics platform coupled with ML.

**Methods:**

Untargeted metabolomics quantification (UHPLC/Q-TOF-MS) was performed for urine analysis. Feature selection was conducted using Lasso regression, and logistic regression, support vector machine, random forest, and extreme gradient boosting were utilized for significance stratification. Pathway enrichment analysis was performed to identify metabolic pathways associated with autism

**Results:**

A total of 52 autistic children and 40 typically developing children were enrolled. Lasso regression identified ninety-two urinary metabolites that significantly differed between the two groups. Distinct metabolites, such as prostaglandin E2, phosphonic acid, lysine, threonine, and phenylalanine, were revealed to be associated with autism through the application of four different ML methods (p<0.05). The alterations observed in the phosphatidylinositol and inositol phosphate metabolism pathways were linked to the pathophysiology of autism (p<0.05).

**Conclusion:**

Significant urinary metabolites, including prostaglandin E2, phosphonic acid, lysine, threonine, and phenylalanine, exhibit associations with autism. Additionally, the involvement of the phosphatidylinositol and inositol phosphate pathways suggests their potential role in the pathophysiology of autism.

## Introduction

Autism spectrum disorder (Autism) is a diverse range of neurodevelopmental disorders characterized by early onset impairments in social interaction and communication, along with stereotyped or repetitive behaviors and restricted interests (1). The worldwide prevalence of autism has shown a significant and rapid increase (2). Despite this, the underlying biological mechanisms remain incompletely understood (3), and the development of precise and effective laboratory diagnostic strategies is lacking. Currently, autism diagnosis heavily relies on clinical interviews and behavioral assessments. The neurodiversity and phenotypic heterogeneity (4) of the condition contribute to the challenges in early diagnosis. Consequently, there is an urgent need for specific diagnostic biomarkers to enhance early screening, diagnosis, and our understanding of the pathophysiology of autism.

Metabolomics, an emerging and promising science field, has proven invaluable in disease diagnosis, elucidating disease processes, and advancing personalized drug targeting and treatments (5). Notably, Metabolomics has played a pivotal role in facilitating swift diagnoses and uncovering unexpected biological underpinnings in cancer and neuropsychiatric disorders (6, 7). The untargeted metabolomics approaches provide a diplomatic avenue for profiling a diverse array of metabolites, offering extensive information for in-depth investigations into disease mechanisms and potential diagnostic biomarkers (8). Exploring and categorizing the metabolic profiles associated with autism holds the potential to deepen our understanding and refine the precision of autism diagnoses. Given the non-independence and high multicollinearity of metabolites, conventional statistical approaches face challenges in effective control (9). ML emerges as a valuable tool in this context, demonstrating its efficacy in accurately identifying significantly differentiated metabolites. ML’s capability to handle high-dimensional, non-independent, and multicollinear metabolomics data enhances our ability to discern intricate patterns in autism-related metabolomic profiles (9).

As such, our aim was to investigate the metabolic profiles of autism using an extensive untargeted metabolomics platform. The objective was to identify distinct metabolites and metabolic pathways associated with autism, thereby fostering exploration and comprehension of the underlying biological mechanisms. The integration of ML further contributes to gaining insights for the development of a predictive or diagnostic tool. This holds promise for enhancing early screening and diagnosis procedures for autism.

## Methods

### Study participants

The autistic children were enrolled at the Neurology Department of Guangzhou Women and Children's Medical Center between September 2018 and October 2020. The typically developing children without a family history of autism, matched by age, were also enrolled from kindergarten in Guangzhou. Inclusion criteria for autistic children were as follows: 1) the Diagnostic and Statistical Manual of Mental Disorders, Fifth Edition (DSM-5) (1). 2) the Autism Diagnostic Observation Schedule (ADOS) (10) and the Autism Diagnostic Interview-Revised (ADI-R) (11), both of which have demonstrated high reliability and validity in diagnosing autism in children. 3) aged between 1 and 14 years, with those diagnosed before two years of age followed up to confirm the diagnosis by at least two years old. 4)Children associated with neurologic, genetic, metabolic, degenerative, or childhood disintegrative disorders were excluded. In addition, demographic information was collected, and the clinical phenotypes of autistic children were assessed. Written consent was obtained for the recruitment of participants. The study was approved by the Guangzhou Women and Children’s Medical Center Ethics Committee (2018031402).

Covariate data were collected at enrollment and included information such as gender, race, age (in years), birth weight (in kilograms), birth height (in centimeters), gestational week, delivery mode, maternal age at birth (in years), and paternal age at birth (in years). Gender was categorized as male or female. Gestational age was divided into preterm (< 37 weeks) and full-term (37 to less than 42 weeks). Delivery mode was categorized as either vaginal birth or cesarean. Additionally, information on gender, race, age, birth weight, birth height, maternal age, and paternal age at birth were treated as continuous variables.

### Sample collection

The urine of the subjects was collected after fasting for more than 4 hours. Following centrifugation, 150μl of each sample was stored in an Eppendorf tube and rapidly frozen at -80°C.

### Untargeted metabolomic analysis

All samples were acquired by the LC-MS auto-sampling system with random orders in the positive and negative ion modes. The analytical conditions were as follows, Ultra-Performance Liquid Chromatography (UPLC): column, Waters ACQUITY UPLC HSS T3 C18 (1.8μm, 2.1 mm*100 mm); column temperature, 35°C; flow rate, 0.3 mL/min; injection volume, 1μL; solvent system, water (0.01% methanolic acid):acetonitrile; gradient program of positive ion, 95:5V/V at 0 min, 79:21 V/V at 3.0 min, 50:50 V/V at 5.0 min,30:70 V/V at 9.0 min, 5:95 V/V at 10.0 min, 95:5 V/V at 14.0 min; gradient program of negative ion, 95:5 V/V at 0 min, 79:21 V/V at 3.0 min, 50:50 V/V at 5.0 min, 30:70 V/V at 9.0 min, 5:95 V/V at 10.0 min, 95:5 V/V at 14.0 min. The Proteo Wizard software transformed the raw data file from LC-MS analysis into mzML format. The XCMS program was manipulated for peak extraction, alignment, and retention time correction. Filter the peaks with a defect rate > 50% in each group of samples. Finally, the metabolic identity information was acquired by matching the laboratory’s self-built database and the public database and metDNA. In the study, quality control (QC) samples (the mixed samples) were inserted into the line to assess the repeatability and reliability of the instrument in every 15 samples. The total ion flow diagram (TIC diagram) of different QC samples presented the instrument’s stability. The higher the overlap of the TIC diagram, the better the stability of the mass chromatogram signal to the same sample at different times.

### Statistical analysis

Following the previous study (12), we meticulously processed the data. The categorical variables underwent comparison through the chi-squared test. Non-normally distributed continuous variables were assessed using the Wilcoxon rank-sum test. Normally distributed continuous variables were compared using the T-test. Employing multivariate statistical analyses, including principal component analysis (PCA), partial least squares discriminant analysis (PLS-DA), and orthogonal partial least squares discriminant analysis (OPLS-DA), we delved into the metabolite profiles distinguishing autistic children from typically developing children. Utilizing criteria such as Fold Change (FC) variations and Variable Importance in Projection (VIP) values (FC≥2/≤ 0.5 and VIP ≥1.0), we identified metabolites that significantly differed between the two groups.

We performed four methods of ML as follows. Initially, Least Absolute Shrinkage and Selection Operator (LASSO) was performed for the feature-selection analysis, leveraging the Lasso to streamline the number of metabolites based on their future importance and address the issue of metabolite multicollinearity. LASSO was a regularization method for linear regression that introduced an L1 regularization term in the loss function. This term encouraged some of the model coefficients to become zero, thereby achieving feature selection. The features selected by LASSO were referred to as important variables. The study employed 10-fold cross-validation to select features from the training set. Values of alpha =0.1 and lambda=0.2 were finally selected as the optimal regularization solution. Subsequently, we utilized logistic regression (LR), support vector machine classification (SVM), random forest (RF), and extreme gradient boosting (XGB) after adjustment for gender, age, birth weight, birth length, gestational week, delivery mode, maternal age at birth and paternal age at birth. The ML models underwent training on 2/3 subsets, while the remaining 1/3 holdout subsets were employed for final model validation. To ensure robustness and guard against overfitting, we conducted a tenfold cross-validation repeated 500 times, thereby corroborating the final results.

The biomarker features of each model were established with a significance threshold of P<0.05 in logistic regression (LR). In SVM, RF, and XGB models, metabolites were considered based on being in the top 10% of the most-weighted features. The final features for the model were selected by consensus, requiring a feature to be identified by at least two modeling approaches. In order to minimize the potential impact of confounding factors as much as possible, the study also adjusted for the following variables such as child gender, child’s age, birth weight, birth length, gestational week, delivery mode, maternal age at birth, and paternal age at birth. We introduced these confounding variables into the model, treating them as analogous to a metabolite modeled alongside the ultimately selected features, eliminating the impact of confounding variables on the results.

**Figure 1:**
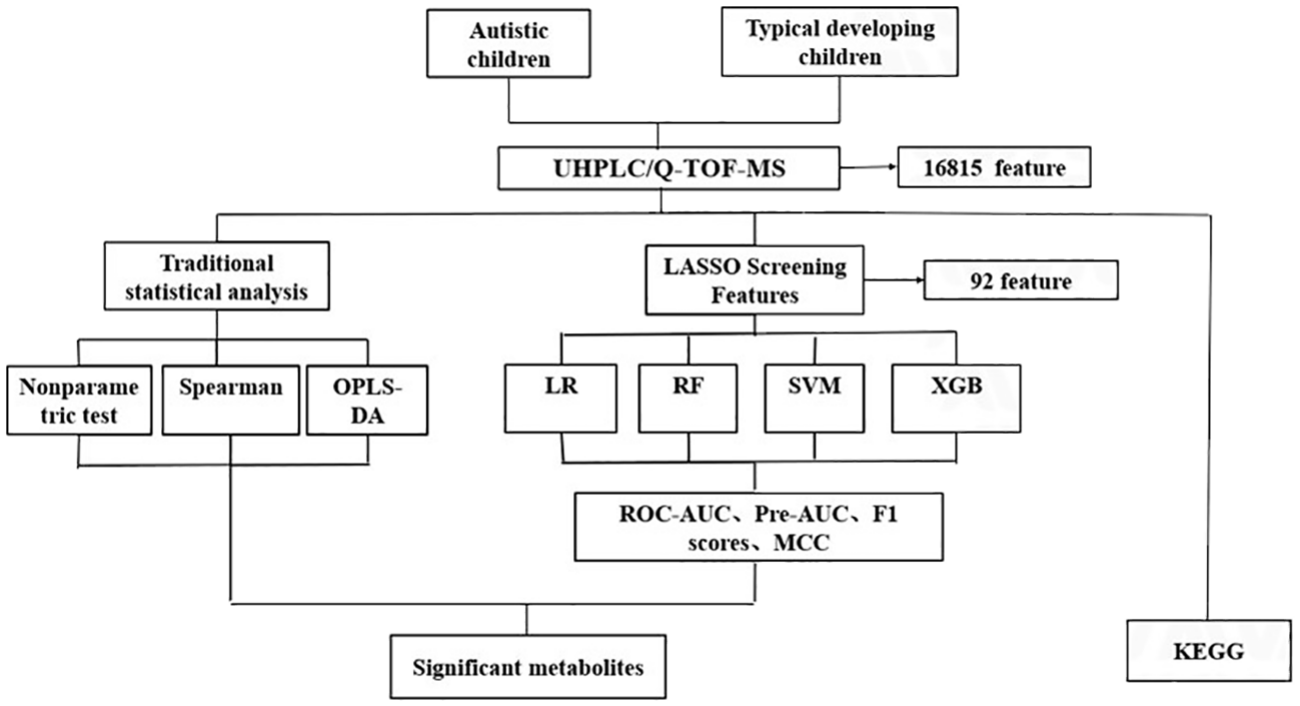
The analytic flow chart for identifying differential metabolites associated with autism. UHPLC/Q-TOF-MS, ultra-high performance liquid chromatography-quadrupole time-of-flight mass spectrometry; OPLS-DA, orthogonal partial least square discriminant analysis; LR, Logistic regression; RF, Random forest; SVM, support vector machine; XGB, Extreme gradient boosting; ROC-AUC, receiver-operator area-under-the-curve; PR-AUC, precision-recall area-under-the-curve; MCC, Matthews correlation coefficient; KEGG, Kyoto Encylopedia of Genes and Genomes.

Additionally, a sensitivity analysis was conducted, wherein ML was employed to identify the most significant metabolites specifically in males. Subsequent to this, pathway enrichment analyses were executed, and significant pathway associations with autism were reported based on a significance threshold of P<0.05. The analytic flow chart is shown in **Figure 1**.

## Result

### The baseline of the study population

A total of 52 autistic and 40 typically developing children were included. Among the 92 children, the median baseline age was 3.52 years, with 84.8% being male,89% identifying as Han ethnicity, 89.1% born at full term, and 67.4% of the children were delivered vaginally. The mean birth weight was 3.27 ± 0.60 kg, and the mean birth height was 49.97 ± 6.08 cm. Maternal age at birth was 30.89 years, while paternal age at birth averaged 32.99 years (**Table 1**). The age distribution of all participants is shown **Figure S1**.

**Table 1:** Baseline characteristics of participants.

Characteristic	case children (n=52)	typical children (n=40)	total (n=92)
male,n(%)^a*^	38(73.1)	40(100.0)	78(84.8)
race,n(%)^a^			
Han	50(96.2)	39(97.5)	89(96.7)
Others	2(3.8)	1(2.5)	3(3.3)
**age,median(IQR)** ^a^ ^*^	2.84(1.95)	3.69(0.44)	3.52(1.22)
**birthweight,mean(SD)** ^c^	3.24(0.57)	3.31(0.63)	3.27(0.60)
**birthlength,mean(SD)** ^c^	50.67(4.84)	49.05(7.35)	49.97(6.08)
Gestational Weeks			
Preterm	3(5.8)	7(17.5)	10(10.9)
fullterm	49(94.2)	33(82.5)	82(89.1)
Delivery Mode^a^			
cesarean delivery	15(28.8)	15(37.5)	30(32.6)
vaginal delivery	37(71.2)	25(62.5)	62(67.4)
**maternal age at birth, median(IQR)** ^b *^	28.74(4.75)	32.61(3.76)	30.89(5.37)
**paternal age at birth, median(IQR)** ^b*^	30.72(5.39)	35.10(4.08)	32.99(6.61)

IQR, interquartile range; SD, standard deviation.

a Categoric variables were compared by a chi-squared test.

b Continuous variables without normal distribution were compared by Wilcoxon rank-sum test.

c Continuous variables with normal distribution were compared by T-test.

^*^There was a significant difference between autistic children and typically developing children, p<0.05.

### Metabolic profiling using untargeted metabolomics

The present study was conducted utilizing an integrated platform capable of detecting 8949 compounds in positive ion mode and 7866 compounds in negative ion mode. Specifically, 186 compounds were examined in positive ion mode, while 181 were examined in the negative ion mode.

**Figure 2:**
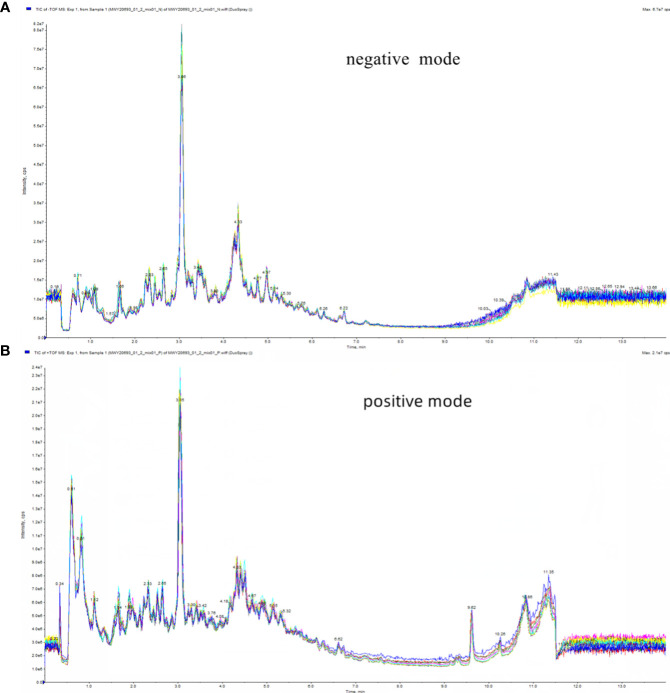
Total ion flow diagram (TIC) of mass chromatograms analysis of quality control samples. **(A)** in positive ion mode; **(B)** in negative ion mode.

In this study, the Total Ion Chromatogram (TIC) diagram from mass chromatogram analysis of quality control samples demonstrated excellent stability of the instrument, ensuring the repeatability and reliability of the data (**Figure 2**). Principal Component Analysis (PCA) assessed the extent of variation and the overall metabolic differences. Notably, in our investigation, the QC samples did not exhibit separation, indicating the robust stability of the instrument (**Figure S2**).

### Identification of differential metabolites by traditional methods

**Figure 3:**
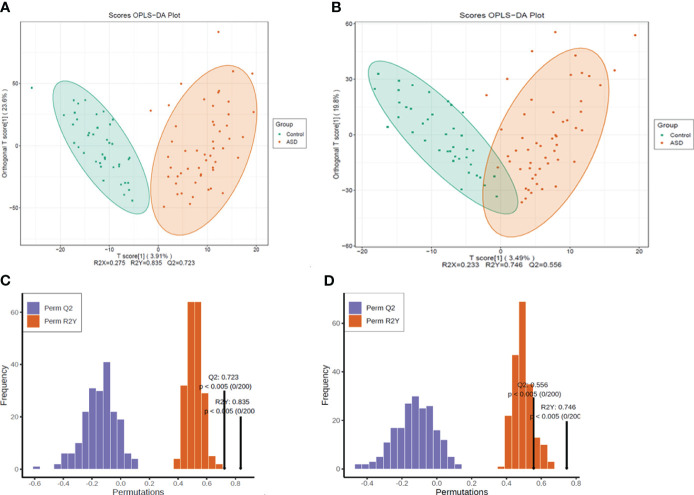
All the detected metabolites were presented as **(A)** OPLS-DA score map, positive ion mode; **(B)** OPLS-DA score map, negative ion mode; **(C)** Permutation test, positive ion mode; **(D)** permutation test, negative ion mode.

The metabolomic data were illustrated through the score map of Partial Least-Discriminant Analysis (OPLS-DA) in both positive and negative ion mode, clearly demonstrating the distinctions between the autism and control group (**Figures 3A, B**). Model permutation tests were conducted in positive ion mode (R2X=0.275, R2Y=0.835, Q2 = 0.723) and negative ion mode (R2X=0.233, R2Y= 0.746, Q2 = 0.556), indicating robustness (**Figures 3C, D**).

**Figure 4:**
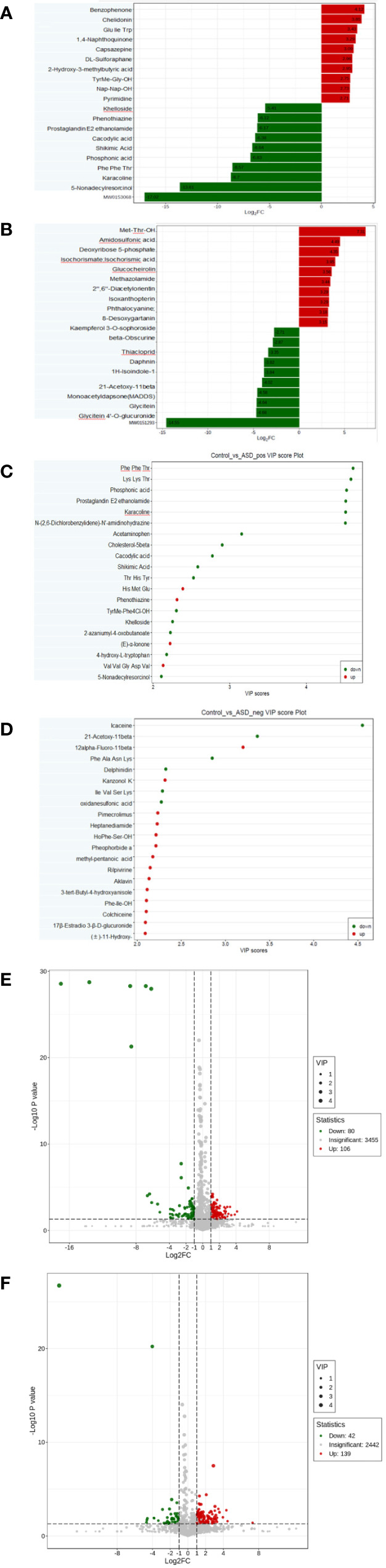
Differential metabolites between autistic children and typically developing children were shown by: **(A)** Fold change, positive ion mode; **(B)** Fold change, negative ion mode; **(C)** VIP value, positive ion mode; **(D)** VIP value, negative ion mode; **(E)** Volcano plot, positive ion mode; **(F)** Volcano plot, negative ion mode.

Among the detected metabolites, our study examined the differential compounds based on both Fold Change (FC) variations (**Figures 4A, B**) and Variable Importance in Projection (VIP) values (**Figures 4C, D**) (FC≥2/≤ 0.5 and VIP ≥1.0). The screening differential metabolites in the positive and negative ion modes were visualized in the volcano map (**Figures 4E, F**).

### Identification of differential metabolites by ML

#### Lasso feature selection

Lasso regression was employed to filter candidate metabolites, resulting in the selection of 92 metabolites (**Table S1**). The various parameters during LASSO feature selection are presented in **Table S2**. Ultimately, we chose alpha=0.1 and lambda=0.2 as the optimal regularization solution.

**Figure 5:**
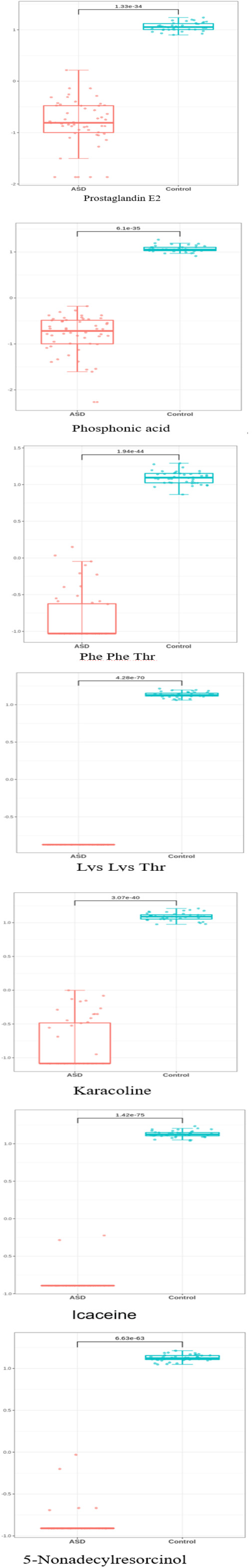
The difference in significant metabolite selected by machine learning between autistic children and typically developing children Y-axis represents Z-score.

The most significant features were chosen based on their significance in three out of the four modeling approaches. Subsequently, our focus narrowed down to seven key metabolites, including Prostaglandin E2, Phosphonic acid, Phenylalanine Phenylalanine Threonine (Phe Phe Thr), Lysine Lysine Threonine (Lys Lys Thr), and others (**Table 2**). The models’ performance was evaluated using four evaluation metrics; four indicators of receiver-operator area-under-the-curve (ROC-AUC), precision-recall area-under-the-curve (PR-AUC), F1 score, and Matthews correlation coefficient (MCC), as presented in **Table 3**. The difference in significant metabolites selected by ML between autistic children and typically developing children was illustrated through Boxplots (**Figure 5**). Sensitivity analyses consistently yielded comparable results, with the most significant metabolites chosen in male participants by ML aligning with those of the entire population. (**Table S3**).

**Table 2:** Implicated metabolites based on multiple modeling approaches.

Metabolite	Class. I	Class. II	VIP	p-value	Fold Change	Approaches Implicated
Lys Lys Thr	–	–	4.526	<0.001	<0.05	LR, RF, SVM
Phosphonic acid	Benzenoids	Benzene and substituted derivatives	4.515	<0.001	<0.05	LR, RF, SVM, XGB
Prostaglandin E2 ethanolamide	Lipids and lipid -like molecules	Fatty Acyls	4.513	<0.001	<0.05	LR, RF, SVM
Phe Phe Thr	–	–	4.582	<0.001	<0.05	LR, RF, SVM
Icaceine	Lipids and lipid -like molecules	Steroids and steroid derivatives	4.553	<0.001	<0.05	LR, RF, SVM
Karacoline	Lipids and lipid -like molecules	Prenol lipids	4.510	<0.001	<0.05	LR, RF, SVM, XGB
5-Nonadecylresorcinol	Benzenoids	Phenols	4.610	<0.001	<0.05	LR, RF, SVM

VIP, variable importance in projection; Lys Lys Thr, Lysine Lysine Threonine; Phe Phe Thr, Phenylalanine Phenylalanine Threonine; LR, logistic regression; RF, random forest; SVM, support vector machine classification; XGB, extreme gradient boosting.

**Table 3:** ML model performance exceeds no-skill prediction in 1/3 holdout subsets based on four evaluation metrics.

Model	ML Model Performance Metric			
	ROC-AUC	PR-AUC	F1 Score	MCC
No-skill	0.5	0.5	0.5	0.5
SVM	1	1	1	1
RF	1	1	1	1
XGB	1	1	1	1

The results were not changed before and after adjusting confounding variables. Adjusted confounding variables included child gender, child’s age, birth weight, birth length, gestational week, delivery mode, maternal age at birth, and paternal age at birth. ROC-AUC may overestimate model performance with a lower case prevalence. PR-AUC excels in disposing of the skewed case distribution. The F1 score is a harmonic mean of precision and recall, with 0 being equivalent to no-skill and 1 being perfect prediction. Similar to the F1, MCC includes true negatives, with-1 being perfect negative prediction and 0 being perfect positive prediction.

LR, logistic regression; RF, random forest; SVM, support vector machine classification; XGB, extreme gradient boosting; ROC-AUC, receiver-operator area-under-the-curve; PR-AUC, precision-recall area-under-the-curve; MCC, Matthews correlation coefficient.

#### KEGG functional enrichment analysis

**Figure 6:**
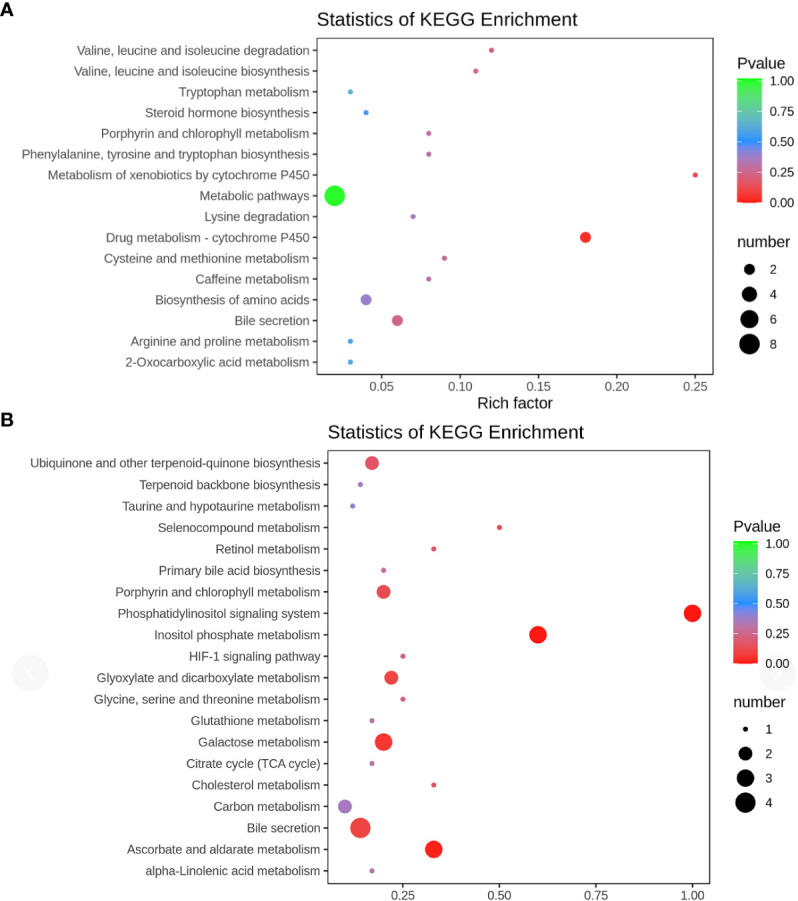
KEGG pathway **(A)** positive ion mode; **(B)** negative ion mode KEGG pathway enrichment was performed based on the differential metabolites. The rich factor is the ratio of the number of differentially expressed metabolites in the corresponding pathway to the total number of metabolites detected and annotated in the pathway. The larger the value, the higher the enrichment degree. The p-value is closer to 0, the enrichment is more significant. The size of the dots represents the number of differentially significant metabolites enriched in the corresponding pathway.

KEGG enrichment analysis was conducted to unveil the metabolic pathways most relevant to autism. Notably, the Phosphatidylinositol signaling system (P = 0.0002) and Inositol phosphate metabolism (P = 0.0002) emerged as identified pathways with potential significance in the context of autism (**Figure 6**)

## Discussion

In this study, machine learning (ML) was utilized to examine urine metabolite profiles in autistic children in comparison to typically developing children. Significantly distinct metabolites, including prostaglandin E2, lysine, and phosphonic acid, were identified as associated with autism. Additionally, KEGG functional enrichment analysis highlighted the significantly perturbed metabolic pathway primarily attributed to phosphatidylinositol and inositol phosphate metabolism.

Our study suggested a potential role for prostaglandin E2 ethanolamide (PGE2-EA) in the pathogenesis of autism. In our study, a potential role for PGE2-EA in autistic children was observed to be lower compared to typically developing children. PGE2-EA is a naturally occurring neutral lipid derivative of prostaglandins synthesized *in vivo* through COX-facilitated oxygenation of arachidonoyl ethanolamine (anandamide) (13). Similar to PGE2, PGE2-EA can bind to all four Prostaglandin E Receptor subtypes (EP1–EP4), implying that PGE2-EA is likely to exhibit similar biological actions compared to PGE2 (14). Additionally, PGE2-EA has been reported to restrain the inflammatory response of human monocytic cells by inhibiting the level of TNF-α (13) and it can be generated by inflammatory cells such as human monocytes. However, further exploration is required to determine if the quantity of PGE2-EA synthesized is pathophysiologically related to the inflammatory conditions observed in autism. While few studies have reported the association between autism and PGE2-EA in urine or blood, previous studies has linked prostaglandin E2 (PGE2) to autism. Qasem L et al (15) observed the highest level of PGE2 in the plasma of neurotypical controls and the lowest level in autistic individuals. They also reported that the lower level of PGE EP2 in plasma was associated with the severity of autism, with a lower PGE EP2 level correlating with lower CARS scores. Furthermore, PGE2 is closely related to the Wnt signaling pathway, which regulates neuronal connectivity and may be implicated in the development of autism (16, 17). PGE2 has also been reported to be associated with the activation of microglia, a process linked to autism (18). Our study is the first to observe lower levels of PGE2-EA in the urine of autistic children compared to healthy controls. However, further investigation is needed to determine whether the biological functions of PGE2-EA are similar to those of PGE2 and whether the unique biological functions of PGE2-EA are related to autism.

Our investigation revealed decreased levels of amino acid metabolites, including lysine, threonine, and phenylalanine, which were found to be associated with autism. Autistic children often experience selective eating habits and gastrointestinal microbiota imbalances, potentially leading to reduced protein intake, and/or inadequate digestion and absorption into amino acids. Additionally, studies have reported a connection between fecal microbiota and abnormal amino acid metabolism, affecting amino acids such as histidine, lysine, tyrosine, phenylalanine, and tryptophan (19–23). Liu A et al. specifically highlighted decreased lysine metabolism in the urine of autistic children (24). Lysine, being an essential amino acid, is susceptible to degradation during processing. Our findings align with previous research showing a decreased level of threonine in the urine of autistic children compared to controls (25). Furthermore, phenylalanine, essential for tyrosine transformation (26), exhibited decreased levels in the plasma of autistic children compared to typically developing children (27, 28). The well-established connection between abnormal phenylalanine metabolism and severe neurological symptoms, as observed in phenylketonuria, a condition often associated with symptoms of autism (29, 30), reinforces our data. Our data suggested a potential association between autism and gut microbiome-derived metabolites. Recent studies have linked abnormal expression of tyrosine and tryptophan to increased disruptive behavior in autistic children without regression (31). The altered levels of amino acid metabolites produced by gut bacteria may serve as potential markers for autism (32). The abnormal amino acid metabolism found in this study provides the opinion for early screening and targeted intervention of autism. Further investigation into the profiles of microbiome-derived metabolites in autism and their role in autism development is essential.

Our study reveals, for the first time, a correlation between the level of phosphatidic acid in urine and autism. Phosphatidic acid is a potent and specific inhibitor of phosphatidylinositol-3 (PI3)-kinase (33), a key player in the PI3K-AKT-MTOR signaling pathway that may regulate the pathogenesis of autism. PI3K activation is crucial for cell division stimulation, apoptosis inhibition, and the proliferation/differentiation of synaptic and neural circuit development from prenatal to early postnatal stages (34). In our investigation, phosphatidic acid emerged as a potential metabolite involved in the etiology of autism. Phosphatidic acid also acts as a potent and specific inhibitor of phospholipase A2 (PLA2), an enzyme vital for maintaining membrane phospholipids. There are three major types of PLA2 enzymes: the calcium-dependent group IV cytosolic PLA2, the group II secretory PLA2, and the group VI calcium-independent PLA2 (35, 36). Increased PLA2 levels in blood have been associated with psychiatric disorders such as autism, depression, and bipolar disorder (37, 38). Previous studies pointed out that there are three single nucleotide polymorphisms in the gene encoding for cytosolic PLA 2 linked to the etiology of schizophrenia (39, 40). The genes encoding human calcium-independent PLA 2 and secretory PLA 2 are associated with autism (41–43). The altered levels of arachidonic acid and DHA in autistic children may be attributed to abnormalities in PLA 2. Altered levels of arachidonic acid and DHA in autistic children may be attributed to abnormalities in PLA2, with elevated activity of type IV PLA2 reported in children with autism and Asperger’s syndrome (37, 44), suggesting an abnormal lipid signaling pathway in autism.

The variations in urinary metabolites indicate differences in metabolic mechanisms between autistic children and typically developing children to some extent. It is noteworthy that this study did not conduct further non-target or targeted analyses of metabolites in serum. Future investigations should aim to validate the expression of differentially screened metabolites in serum between autistic children and typically developing children. Differential metabolites and their downstream metabolites, such as prostaglandin E2, and dopamine, can significantly impact brain development by crossing the blood-brain barrier (45, 46). Phenylalanine, crucial for the transformation of tyrosine, serves as a precursor to dopamine, which is associated with ASD development (27, 28, 45). Prostaglandin E2 acts as a vital signaling molecule, exerting its effects through the activation of respective G-protein-coupled receptors (46). The prostaglandin E2 played important roles in neurodevelopment including synaptic plasticity and long-term potentiation or inflammation (47). Arachidonic acid, a precursor of key lipid mediators like prostaglandin E2, is released by PLA 2 from the sn-2 position of phospholipids (35, 36), and PLA 2 is considered to play a pivotal role in neurodevelopment (48).

In addition, karacoline, icaceine, and 5-Nonadecylresorcinol were also identified as differential metabolites between autistic children and typically developing children. However, the role of these metabolites in autism based on experimental studies remains poorly documented. Future investigations are warranted to explore the connection between these metabolites and the development of autism.

### KEGG functional enrichment

The present study first suggested that the alterations of the phosphatidylinositol signaling pathway and the inositol phosphate metabolism pathway might contribute to the pathophysiology of autism. The phosphoinositide signaling pathway and myoinositol were also reported to be associated with epilepsy by the action on gamma amino butyric acid-A receptors (49). Recent literature reported that epilepsy was one of the most common comorbidities of autism and the updated pooled prevalence of epilepsy in autistic individuals was 10% (95% CI: 6%–14%) (50). Our findings indicated that there might be a possible pathway for autism and epilepsy. In the present study, we have also identified the increased inositol 1,3-bisphosphate (1,3-IP 2) and myo-Inositol(I) of urine of autistic children, which are associated with the phosphatidylinositol signaling pathway and the inositol phosphate metabolism pathway. Consistent with our findings, recent studies have reported an elevated myo-inositol level in the autism group compared to the typically developing (TD) group (51, 52). The autism spectrum disorder group demonstrated significantly higher myo-inositol/creatine ratios in the hippocampus-amygdala and cerebellar regions (53). In the TD group, myo-inositol/creatine ratios in the left and right hippocampus-amygdalas exhibited an inverse relationship with performance, verbal, and full-scale IQ scores (53). Myo-inositol, primarily located in astrocytes, is associated with high myo-inositol/creatine ratios, indicating an abnormal condition characterized by increased glial cells or myelin degradation (54). Therefore, the higher myo-inositol level may explain increased overall cellular growth and size in the right hippocampus-amygdala of autistic children (53). The upstream metabolites of IP3 are respective 1,3,4-bisphosphate (1,3,4-IP3) and inositol 1,3,4,5-bisphosphate (1,3,4,5-IP4), while the downstream are inositol 1-phosphate (1-IP) and myo-Inositol (I) (55, 56). These metabolisms involved the PI3K-AKT-MTOR signaling pathway (57, 58). Previous studies demonstrated that the PI3K-AKT-MTOR signaling pathway might regulate the pathogenesis of autism (59, 60).

## Strength and limitation

This study represents the first comprehensive exploration of the metabolic profiles in the urine of autistic children utilizing ML. ML, adept at handling high-dimensional, non-independent, and multicollinear metabolomics data, played a crucial role in identifying robust associations between differential metabolites and autism. Pathway enrichment analyses were incorporated to recognize associated pathways rather than individual metabolites, leveraging the multicollinearity of metabolites. Additionally, the diagnosis of autistic children adhered to DSM-5 criteria by two neuropsychiatrists, complemented by interviews with ADOS and ADI-R. Furthermore, urine samples were collected at the diagnostic point from the autistic children cohort, promptly divided, and frozen at -80°C to mitigate the potential impact of repeated freeze-thaw cycles, thereby enhancing the study’s validity.

Despite these strengths, several limitations merit consideration. Firstly, the observational nature of the study introduces the possibility of residual or unmeasured confounding factors influencing the associations between differential metabolites and autism. Efforts were made to adjust for confounding variables, and the results exhibited good consistency post-adjustment. Secondly, the observational design restricts causal inference. Thirdly, the absence of females in the typically developing children cohort is a notable limitation. However, a sensitivity analysis, including only male autistic children, yielded consistent results. Fourthly, the lack of a validation set in the study prevents the independent verification of results. Fifthly, the small sample size may limit statistical power, emphasizing the need for large-scale population studies to validate the findings. Lastly, there was a discordance in age distribution between autistic children and typically developing children.

## Conclusion

The present study unveiled a spectrum of dysmetabolism profiles potentially implicated in autism, potentially implicated in autism prostaglandin E2, phosphonic acid, and so on. The alterations of the phosphatidylinositol and the Inositol phosphate pathway may contribute to the pathophysiology of autism. Leveraging metabolomics and novel analytical approaches not only sheds light on these dysmetabolic profiles but also paves the way for future investigations aimed at advancing early screening, precise diagnosis, and a deeper understanding of the pathophysiological mechanisms underlying autism.

## Data availability statement

The original contributions presented in the study are included in the article/**Supplementary Material**. Further inquiries can be directed to the corresponding authors upon reasonable request.

## Ethics statement

The studies involving humans were approved by Guangzhou Women and Children’s Medical Center Ethics Committee (2018031402). The studies were conducted in accordance with the local legislation and institutional requirements. Written informed consent for participation in this study was provided by the participants’ legal guardians/next of kin.

## Author contributions

XL: Validation, Writing – review & editing, Data curation, Formal analysis, Methodology, Software, Writing – original draft. XS: Data curation, Formal analysis, Methodology, Software, Validation, Writing – review & editing, Investigation. CG: Investigation, Formal analysis, Software, visualization, Writing – review & editing. HZ-F: Data curation, Writing – original draft, Investigation. YC: Data curation, Writing – original draft. F-MF: Data curation, Writing – original draft. LW: Conceptualization, Resources, Supervision, Validation, Visualization, Writing – review & editing. W-XC: Conceptualization, Resources, Supervision, Validation, Visualization, Writing – review & editing, Funding acquisition, Project administration.
