# Supplementary material for: Untargeted urine metabolomics and machine learning provide potential metabolic signatures in children with autism spectrum disorder
Source: Front Psychiatry. 2024 Feb 20;15:1261617. doi: 10.3389/fpsyt.2024.1261617 (PMC10912307; doi:10.3389/fpsyt.2024.1261617)
Supplement: Supplementary file 1 [file DataSheet_1.docx]

**TableS1 The total of 92 metabolites selected by LASSO regression**

| **metabolites** | P_Value |
| --- | --- |
| Phe Phe Thr | 5.144102104706559e-22 |
| 4-(Acetylamino)-N-(2,6-dimethylphenyl)benzamide | 0.32140064806914426 |
| Phenothiazine | 0.0005976362198493646 |
| Valeric acid ethyl ester | 4.7081675715574656e-11 |
| Daphnin | 0.012592107733611841 |
| Monocrotaline | 2.1383838325649413e-15 |
| 12alpha-Fluoro-11beta,17beta-dihydroxyandrosta-1,4-dien-3-one | 3.192667566637696e-08 |
| N-(2,6-Dichlorobenzylidene)-N'-amidinohydrazine | 1.849697371536844e-08 |
| Acetaminophen | 7.907463433740617e-07 |
| Lucidenic acid B | 1.7695079868193812e-17 |
| Laricitrin 3-galactoside | 1.9529217479298012e-13 |
| Lys Phe Asn | 1.891389073579392e-13 |
| Asp-Tyr-OH | 0.16835975804596826 |
| Oxyphenbutazone | 1.6625611688382288e-13 |
| Sulindac sulfone | 0.004592082651480487 |
| Clobenpropit | 0.0077434813754249845 |
| Prostaglandin A1 ethyl ester | 7.223850795005349e-14 |
| Levocetirizine | 0.5295613590166048 |
| Tyr Arg Ile | 2.5544526643240197e-13 |
| 17-keto-4(Z),7(Z),10(Z),13(Z),15(E),19(Z)-Docosahexaenoic Acid | 1.5898051768572928e-11 |
| Arachidonoyl-N,N-dimethylamide | 2.267766565168425e-08 |
| Procyanidin C1 | 1.7473028824821873e-11 |
| 17a,20a-Dihydroxycholesterol | 1.5528084903147014e-05 |
| 1-Heptanol | 6.836689610727598e-12 |
| TG(16:1(9Z)/18:2(9Z,12Z)/18:1(11Z)) | 1.0120776610814177e-09 |
| 4-Hydroxy-3-methoxy-5-(benzothiazolylthiomethyl)benzylidenecyanoacetamide | 0.3088960923998333 |
| Sclareolide | 3.0232009126767227e-09 |
| TREMULACIN | 0.06218697986504924 |
| Alprenolol | 1.1532580663452049e-14 |
| Cyclosulfamuron | 0.14702717964213372 |
| Mallotochromene | 0.3217629696759856 |
| Phe Ala Asn Lys | 0.0001276589700047279 |
| Tetrahydromagnolol | 3.1271257419738014e-07 |
| Lys Met Val Ser Arg | 0.0017533548295693958 |
| Arg Ile Asp | 1.1372852781162638e-09 |
| 2-Hydroxyquinoline | 0.012476581783986418 |
| 3-(3-hydroxy-4-methoxyphenyl)prop-2-enoic acid | 1.7733830488902597e-08 |
| N-Arachidonoyl dopamine | 8.921551359455725e-15 |
| Bufalin | 4.802709324019575e-11 |
| Asn Thr Gln Glu | 0.06687831111889155 |
| 3-Epiecdysone | 9.958143582157159e-06 |
| Diisooctyl phthalate | 1.3762761494883585e-14 |
| 1,3-Dicyclohexylurea | 1.1920100187910172e-10 |
| Cholesterol-5beta,6beta-epoxide;5beta,6beta-Epoxycholestan-3beta-ol;5,6beta-Epoxy-5beta-cholestan-3beta-ol | 1.2043358947644905e-05 |
| Val Ser Gly Pro Glu | 0.16537882974555748 |
| 10-Propoxydecanoic acid | 0.018437211422537233 |
| Tricosanoic acid | 1.6583292357227707e-07 |
| Lys Val Thr | 0.0001340320516737046 |
| D-Fructose 6-phosphate | 0.7617520839631519 |
| 3-Hydroxy-3-methylbutanoic acid | 0.007345428665507777 |
| Chaksine | 8.390038868697472e-10 |
| Avermitilol | 1.8909523668419668e-08 |
| BETAINE | 4.836475771268328e-07 |
| 2,3-Dihydroxypropanal | 0.017534331359715882 |
| Arg Gln Ser Lys | 0.0017148634051847064 |
| LysoPA(16:0/0:0) | 1.939031613475789e-10 |
| stigmasta-7,22E-dien-3 -ol | 6.868707475491518e-11 |
| [5,7-dihydroxy-2-(4-hydroxy-3-methoxyphenyl)-4-oxo-3,4-dihydro-2H-1-benzopyran-3-yl]oxidanesulfonic acid | 0.08953828651768239 |
| 9-Oxo-11-(3-pentyl-2-oxiranyl)-10E-undecenoic acid | 4.630895017573413e-07 |
| Lys Arg Phe | 4.1329328110092195e-09 |
| 1-Hydroxy-1-(4-methoxyphenyl)propan-2-yl hexopyranoside | 0.5311096372075466 |
| 3',4',7-Trihydroxyflavone | 0.0954490741873708 |
| N-3-oxo-hexadec-11(Z)-enoyl-L-Homoserine lactone | 0.0002534219194565772 |
| N'-(4-Methoxybenzoyl)-2-{[4-methyl-5-(3-pyridinyl)-4H-1,2,4-triazol-3-yl]sulfanyl}acetohydrazide | 1.8858670187509373e-06 |
| Apigenin-7-O-neohesperidoside | 0.07537538758591561 |
| 2-Linoleoylglycerol | 2.3466741983588247e-09 |
| Lys Met His | 2.102980006774246e-06 |
| Cefixime trihydrate | 0.3432872502307437 |
| Polygodial | 0.0018849260011996878 |
| Bepridil | 1.2896167815847035e-07 |
| 7-Hydroxycoumarin | 0.28552111855777945 |
| 1-Arachidonoylglycerol | 5.795079491990502e-06 |
| L-Serine-phosphoethanolamine;Serine phosphoethanolamine | 0.00017683180912022003 |
| Penoxsulam | 0.04602412028619266 |
| N1-Acetylspermidine | 1.6902498649096915e-10 |
| [Nitrilotris(methylene)]trisphosphonic acid | 1.238081010392811e-05 |
| DL-Ephedrine | 0.2681822707786977 |
| Benzophenone | 0.00643740612124815 |
| Cys Met Asp | 0.3218594214062932 |
| Estrone sulfate | 0.00020581396280759773 |
| 5(S),6(R),15(R)-Lipoxin A4 | 1.9304754858819253e-09 |
| 2,4-Quinolinediol | 0.0808830434921675 |
| Lys Glu Ser Leu Ser | 5.076018617660821e-08 |
| beta-Obscurine | 0.0014657528797756513 |
| Diacetyl-10-gingerdiol | 1.3937801741496629e-08 |
| Asn Cys Gly | 0.0016830370831209026 |
| Phe-Pro | 3.362008753590164e-06 |
| Avocadene acetate | 2.7032781576216088e-05 |
| Met-Thr-OH | 0.03827699221025038 |
| 1,2-Dipalmitoyl-sn-glycero-3-phosphoethanolamine-N,N-dimethyl | 0.0005682989329517697 |
| (R)-2-Hydroxy-3-phenylpropionic acid | 3.1452970828199385e-06 |
| Methyl trans-4-oxo-2-pentenoate | 0.003759025277163015 |

**TableS2 The parameters of Lasso regression in cross-validation**

| **alpha** | **lambda** | **Accuracy** | **Kappa** | **AccuracySD** | **KappaSD** |
| --- | --- | --- | --- | --- | --- |
| 0 | 0.002 | 0.973809524 | 0.946376812 | 0.05976143 | 0.122097353 |
| 0 | 0.004 | 0.973809524 | 0.946376812 | 0.05976143 | 0.122097353 |
| 0 | 0.006 | 0.973809524 | 0.946376812 | 0.05976143 | 0.122097353 |
| 0 | 0.008 | 0.973809524 | 0.946376812 | 0.05976143 | 0.122097353 |
| 0 | 0.01 | 0.973809524 | 0.946376812 | 0.05976143 | 0.122097353 |
| 0 | 0.012 | 0.973809524 | 0.946376812 | 0.05976143 | 0.122097353 |
| 0 | 0.014 | 0.973809524 | 0.946376812 | 0.05976143 | 0.122097353 |
| 0 | 0.016 | 0.973809524 | 0.946376812 | 0.05976143 | 0.122097353 |
| 0 | 0.018 | 0.973809524 | 0.946376812 | 0.05976143 | 0.122097353 |
| 0 | 0.02 | 0.973809524 | 0.946376812 | 0.05976143 | 0.122097353 |
| 0 | 0.022 | 0.973809524 | 0.946376812 | 0.05976143 | 0.122097353 |
| 0 | 0.024 | 0.973809524 | 0.946376812 | 0.05976143 | 0.122097353 |
| 0 | 0.026 | 0.973809524 | 0.946376812 | 0.05976143 | 0.122097353 |
| 0 | 0.028 | 0.973809524 | 0.946376812 | 0.05976143 | 0.122097353 |
| 0 | 0.03 | 0.973809524 | 0.946376812 | 0.05976143 | 0.122097353 |
| 0 | 0.032 | 0.973809524 | 0.946376812 | 0.05976143 | 0.122097353 |
| 0 | 0.034 | 0.973809524 | 0.946376812 | 0.05976143 | 0.122097353 |
| 0 | 0.036 | 0.973809524 | 0.946376812 | 0.05976143 | 0.122097353 |
| 0 | 0.038 | 0.973809524 | 0.946376812 | 0.05976143 | 0.122097353 |
| 0 | 0.04 | 0.973809524 | 0.946376812 | 0.05976143 | 0.122097353 |
| 0 | 0.042 | 0.973809524 | 0.946376812 | 0.05976143 | 0.122097353 |
| 0 | 0.044 | 0.973809524 | 0.946376812 | 0.05976143 | 0.122097353 |
| 0 | 0.046 | 0.973809524 | 0.946376812 | 0.05976143 | 0.122097353 |
| 0 | 0.048 | 0.973809524 | 0.946376812 | 0.05976143 | 0.122097353 |
| 0 | 0.05 | 0.973809524 | 0.946376812 | 0.05976143 | 0.122097353 |
| 0 | 0.052 | 0.973809524 | 0.946376812 | 0.05976143 | 0.122097353 |
| 0 | 0.054 | 0.973809524 | 0.946376812 | 0.05976143 | 0.122097353 |
| 0 | 0.056 | 0.973809524 | 0.946376812 | 0.05976143 | 0.122097353 |
| 0 | 0.058 | 0.973809524 | 0.946376812 | 0.05976143 | 0.122097353 |
| 0 | 0.06 | 0.973809524 | 0.946376812 | 0.05976143 | 0.122097353 |
| 0 | 0.062 | 0.973809524 | 0.946376812 | 0.05976143 | 0.122097353 |
| 0 | 0.064 | 0.973809524 | 0.946376812 | 0.05976143 | 0.122097353 |
| 0 | 0.066 | 0.973809524 | 0.946376812 | 0.05976143 | 0.122097353 |
| 0 | 0.068 | 0.973809524 | 0.946376812 | 0.05976143 | 0.122097353 |
| 0 | 0.07 | 0.973809524 | 0.946376812 | 0.05976143 | 0.122097353 |
| 0 | 0.072 | 0.973809524 | 0.946376812 | 0.05976143 | 0.122097353 |
| 0 | 0.074 | 0.973809524 | 0.946376812 | 0.05976143 | 0.122097353 |
| 0 | 0.076 | 0.973809524 | 0.946376812 | 0.05976143 | 0.122097353 |
| 0 | 0.078 | 0.973809524 | 0.946376812 | 0.05976143 | 0.122097353 |
| 0 | 0.08 | 0.973809524 | 0.946376812 | 0.05976143 | 0.122097353 |
| 0 | 0.082 | 0.973809524 | 0.946376812 | 0.05976143 | 0.122097353 |
| 0 | 0.084 | 0.973809524 | 0.946376812 | 0.05976143 | 0.122097353 |
| 0 | 0.086 | 0.973809524 | 0.946376812 | 0.05976143 | 0.122097353 |
| 0 | 0.088 | 0.973809524 | 0.946376812 | 0.05976143 | 0.122097353 |
| 0 | 0.09 | 0.973809524 | 0.946376812 | 0.05976143 | 0.122097353 |
| 0 | 0.092 | 0.973809524 | 0.946376812 | 0.05976143 | 0.122097353 |
| 0 | 0.094 | 0.973809524 | 0.946376812 | 0.05976143 | 0.122097353 |
| 0 | 0.096 | 0.973809524 | 0.946376812 | 0.05976143 | 0.122097353 |
| 0 | 0.098 | 0.973809524 | 0.946376812 | 0.05976143 | 0.122097353 |
| 0 | 0.1 | 0.973809524 | 0.946376812 | 0.05976143 | 0.122097353 |
| 0.1 | 0.002 | 1 | 1 | 0 | 0 |
| 0.1 | 0.004 | 1 | 1 | 0 | 0 |
| 0.1 | 0.006 | 1 | 1 | 0 | 0 |
| 0.1 | 0.008 | 1 | 1 | 0 | 0 |
| 0.1 | 0.01 | 1 | 1 | 0 | 0 |
| 0.1 | 0.012 | 1 | 1 | 0 | 0 |
| 0.1 | 0.014 | 1 | 1 | 0 | 0 |
| 0.1 | 0.016 | 1 | 1 | 0 | 0 |
| 0.1 | 0.018 | 1 | 1 | 0 | 0 |
| 0.1 | 0.02 | 1 | 1 | 0 | 0 |
| 0.1 | 0.022 | 1 | 1 | 0 | 0 |
| 0.1 | 0.024 | 1 | 1 | 0 | 0 |
| 0.1 | 0.026 | 1 | 1 | 0 | 0 |
| 0.1 | 0.028 | 1 | 1 | 0 | 0 |
| 0.1 | 0.03 | 1 | 1 | 0 | 0 |
| 0.1 | 0.032 | 1 | 1 | 0 | 0 |
| 0.1 | 0.034 | 1 | 1 | 0 | 0 |
| 0.1 | 0.036 | 1 | 1 | 0 | 0 |
| 0.1 | 0.038 | 1 | 1 | 0 | 0 |
| 0.1 | 0.04 | 1 | 1 | 0 | 0 |
| 0.1 | 0.042 | 1 | 1 | 0 | 0 |
| 0.1 | 0.044 | 1 | 1 | 0 | 0 |
| 0.1 | 0.046 | 1 | 1 | 0 | 0 |
| 0.1 | 0.048 | 1 | 1 | 0 | 0 |
| 0.1 | 0.05 | 1 | 1 | 0 | 0 |
| 0.1 | 0.052 | 1 | 1 | 0 | 0 |
| 0.1 | 0.054 | 1 | 1 | 0 | 0 |
| 0.1 | 0.056 | 1 | 1 | 0 | 0 |
| 0.1 | 0.058 | 1 | 1 | 0 | 0 |
| 0.1 | 0.06 | 1 | 1 | 0 | 0 |
| 0.1 | 0.062 | 1 | 1 | 0 | 0 |
| 0.1 | 0.064 | 1 | 1 | 0 | 0 |
| 0.1 | 0.066 | 1 | 1 | 0 | 0 |
| 0.1 | 0.068 | 1 | 1 | 0 | 0 |
| 0.1 | 0.07 | 1 | 1 | 0 | 0 |
| 0.1 | 0.072 | 1 | 1 | 0 | 0 |
| 0.1 | 0.074 | 1 | 1 | 0 | 0 |
| 0.1 | 0.076 | 1 | 1 | 0 | 0 |
| 0.1 | 0.078 | 1 | 1 | 0 | 0 |
| 0.1 | 0.08 | 1 | 1 | 0 | 0 |
| 0.1 | 0.082 | 1 | 1 | 0 | 0 |
| 0.1 | 0.084 | 1 | 1 | 0 | 0 |
| 0.1 | 0.086 | 1 | 1 | 0 | 0 |
| 0.1 | 0.088 | 1 | 1 | 0 | 0 |
| 0.1 | 0.09 | 1 | 1 | 0 | 0 |
| 0.1 | 0.092 | 1 | 1 | 0 | 0 |
| 0.1 | 0.094 | 1 | 1 | 0 | 0 |
| 0.1 | 0.096 | 1 | 1 | 0 | 0 |
| 0.1 | 0.098 | 1 | 1 | 0 | 0 |
| 0.1 | 0.1 | 1 | 1 | 0 | 0 |
| 0.2 | 0.002 | 1 | 1 | 0 | 0 |
| 0.2 | 0.004 | 1 | 1 | 0 | 0 |
| 0.2 | 0.006 | 1 | 1 | 0 | 0 |
| 0.2 | 0.008 | 1 | 1 | 0 | 0 |
| 0.2 | 0.01 | 1 | 1 | 0 | 0 |
| 0.2 | 0.012 | 1 | 1 | 0 | 0 |
| 0.2 | 0.014 | 1 | 1 | 0 | 0 |
| 0.2 | 0.016 | 1 | 1 | 0 | 0 |
| 0.2 | 0.018 | 1 | 1 | 0 | 0 |
| 0.2 | 0.02 | 1 | 1 | 0 | 0 |
| 0.2 | 0.022 | 1 | 1 | 0 | 0 |
| 0.2 | 0.024 | 1 | 1 | 0 | 0 |
| 0.2 | 0.026 | 1 | 1 | 0 | 0 |
| 0.2 | 0.028 | 1 | 1 | 0 | 0 |
| 0.2 | 0.03 | 1 | 1 | 0 | 0 |
| 0.2 | 0.032 | 1 | 1 | 0 | 0 |
| 0.2 | 0.034 | 1 | 1 | 0 | 0 |
| 0.2 | 0.036 | 1 | 1 | 0 | 0 |
| 0.2 | 0.038 | 1 | 1 | 0 | 0 |
| 0.2 | 0.04 | 1 | 1 | 0 | 0 |
| 0.2 | 0.042 | 1 | 1 | 0 | 0 |
| 0.2 | 0.044 | 1 | 1 | 0 | 0 |
| 0.2 | 0.046 | 1 | 1 | 0 | 0 |
| 0.2 | 0.048 | 1 | 1 | 0 | 0 |
| 0.2 | 0.05 | 1 | 1 | 0 | 0 |
| 0.2 | 0.052 | 1 | 1 | 0 | 0 |
| 0.2 | 0.054 | 1 | 1 | 0 | 0 |
| 0.2 | 0.056 | 1 | 1 | 0 | 0 |
| 0.2 | 0.058 | 1 | 1 | 0 | 0 |
| 0.2 | 0.06 | 1 | 1 | 0 | 0 |
| 0.2 | 0.062 | 1 | 1 | 0 | 0 |
| 0.2 | 0.064 | 1 | 1 | 0 | 0 |
| 0.2 | 0.066 | 1 | 1 | 0 | 0 |
| 0.2 | 0.068 | 1 | 1 | 0 | 0 |
| 0.2 | 0.07 | 1 | 1 | 0 | 0 |
| 0.2 | 0.072 | 1 | 1 | 0 | 0 |
| 0.2 | 0.074 | 1 | 1 | 0 | 0 |
| 0.2 | 0.076 | 1 | 1 | 0 | 0 |
| 0.2 | 0.078 | 1 | 1 | 0 | 0 |
| 0.2 | 0.08 | 1 | 1 | 0 | 0 |
| 0.2 | 0.082 | 1 | 1 | 0 | 0 |
| 0.2 | 0.084 | 1 | 1 | 0 | 0 |
| 0.2 | 0.086 | 1 | 1 | 0 | 0 |
| 0.2 | 0.088 | 1 | 1 | 0 | 0 |
| 0.2 | 0.09 | 1 | 1 | 0 | 0 |
| 0.2 | 0.092 | 1 | 1 | 0 | 0 |
| 0.2 | 0.094 | 1 | 1 | 0 | 0 |
| 0.2 | 0.096 | 1 | 1 | 0 | 0 |
| 0.2 | 0.098 | 1 | 1 | 0 | 0 |
| 0.2 | 0.1 | 1 | 1 | 0 | 0 |
| 0.3 | 0.002 | 1 | 1 | 0 | 0 |
| 0.3 | 0.004 | 1 | 1 | 0 | 0 |
| 0.3 | 0.006 | 1 | 1 | 0 | 0 |
| 0.3 | 0.008 | 1 | 1 | 0 | 0 |
| 0.3 | 0.01 | 1 | 1 | 0 | 0 |
| 0.3 | 0.012 | 1 | 1 | 0 | 0 |
| 0.3 | 0.014 | 1 | 1 | 0 | 0 |
| 0.3 | 0.016 | 1 | 1 | 0 | 0 |
| 0.3 | 0.018 | 1 | 1 | 0 | 0 |
| 0.3 | 0.02 | 1 | 1 | 0 | 0 |
| 0.3 | 0.022 | 1 | 1 | 0 | 0 |
| 0.3 | 0.024 | 1 | 1 | 0 | 0 |
| 0.3 | 0.026 | 1 | 1 | 0 | 0 |
| 0.3 | 0.028 | 1 | 1 | 0 | 0 |
| 0.3 | 0.03 | 1 | 1 | 0 | 0 |
| 0.3 | 0.032 | 1 | 1 | 0 | 0 |
| 0.3 | 0.034 | 1 | 1 | 0 | 0 |
| 0.3 | 0.036 | 1 | 1 | 0 | 0 |
| 0.3 | 0.038 | 1 | 1 | 0 | 0 |
| 0.3 | 0.04 | 1 | 1 | 0 | 0 |
| 0.3 | 0.042 | 1 | 1 | 0 | 0 |
| 0.3 | 0.044 | 1 | 1 | 0 | 0 |
| 0.3 | 0.046 | 1 | 1 | 0 | 0 |
| 0.3 | 0.048 | 1 | 1 | 0 | 0 |
| 0.3 | 0.05 | 1 | 1 | 0 | 0 |
| 0.3 | 0.052 | 1 | 1 | 0 | 0 |
| 0.3 | 0.054 | 1 | 1 | 0 | 0 |
| 0.3 | 0.056 | 1 | 1 | 0 | 0 |
| 0.3 | 0.058 | 1 | 1 | 0 | 0 |
| 0.3 | 0.06 | 1 | 1 | 0 | 0 |
| 0.3 | 0.062 | 1 | 1 | 0 | 0 |
| 0.3 | 0.064 | 1 | 1 | 0 | 0 |
| 0.3 | 0.066 | 1 | 1 | 0 | 0 |
| 0.3 | 0.068 | 1 | 1 | 0 | 0 |
| 0.3 | 0.07 | 1 | 1 | 0 | 0 |
| 0.3 | 0.072 | 1 | 1 | 0 | 0 |
| 0.3 | 0.074 | 1 | 1 | 0 | 0 |
| 0.3 | 0.076 | 1 | 1 | 0 | 0 |
| 0.3 | 0.078 | 1 | 1 | 0 | 0 |
| 0.3 | 0.08 | 1 | 1 | 0 | 0 |
| 0.3 | 0.082 | 1 | 1 | 0 | 0 |
| 0.3 | 0.084 | 1 | 1 | 0 | 0 |
| 0.3 | 0.086 | 1 | 1 | 0 | 0 |
| 0.3 | 0.088 | 1 | 1 | 0 | 0 |
| 0.3 | 0.09 | 1 | 1 | 0 | 0 |
| 0.3 | 0.092 | 1 | 1 | 0 | 0 |
| 0.3 | 0.094 | 1 | 1 | 0 | 0 |
| 0.3 | 0.096 | 1 | 1 | 0 | 0 |
| 0.3 | 0.098 | 1 | 1 | 0 | 0 |
| 0.3 | 0.1 | 1 | 1 | 0 | 0 |
| 0.4 | 0.002 | 1 | 1 | 0 | 0 |
| 0.4 | 0.004 | 1 | 1 | 0 | 0 |
| 0.4 | 0.006 | 1 | 1 | 0 | 0 |
| 0.4 | 0.008 | 1 | 1 | 0 | 0 |
| 0.4 | 0.01 | 1 | 1 | 0 | 0 |
| 0.4 | 0.012 | 1 | 1 | 0 | 0 |
| 0.4 | 0.014 | 1 | 1 | 0 | 0 |
| 0.4 | 0.016 | 1 | 1 | 0 | 0 |
| 0.4 | 0.018 | 1 | 1 | 0 | 0 |
| 0.4 | 0.02 | 1 | 1 | 0 | 0 |
| 0.4 | 0.022 | 1 | 1 | 0 | 0 |
| 0.4 | 0.024 | 1 | 1 | 0 | 0 |
| 0.4 | 0.026 | 1 | 1 | 0 | 0 |
| 0.4 | 0.028 | 1 | 1 | 0 | 0 |
| 0.4 | 0.03 | 1 | 1 | 0 | 0 |
| 0.4 | 0.032 | 1 | 1 | 0 | 0 |
| 0.4 | 0.034 | 1 | 1 | 0 | 0 |
| 0.4 | 0.036 | 1 | 1 | 0 | 0 |
| 0.4 | 0.038 | 1 | 1 | 0 | 0 |
| 0.4 | 0.04 | 1 | 1 | 0 | 0 |
| 0.4 | 0.042 | 1 | 1 | 0 | 0 |
| 0.4 | 0.044 | 1 | 1 | 0 | 0 |
| 0.4 | 0.046 | 1 | 1 | 0 | 0 |
| 0.4 | 0.048 | 1 | 1 | 0 | 0 |
| 0.4 | 0.05 | 1 | 1 | 0 | 0 |
| 0.4 | 0.052 | 1 | 1 | 0 | 0 |
| 0.4 | 0.054 | 1 | 1 | 0 | 0 |
| 0.4 | 0.056 | 1 | 1 | 0 | 0 |
| 0.4 | 0.058 | 1 | 1 | 0 | 0 |
| 0.4 | 0.06 | 1 | 1 | 0 | 0 |
| 0.4 | 0.062 | 1 | 1 | 0 | 0 |
| 0.4 | 0.064 | 1 | 1 | 0 | 0 |
| 0.4 | 0.066 | 1 | 1 | 0 | 0 |
| 0.4 | 0.068 | 1 | 1 | 0 | 0 |
| 0.4 | 0.07 | 1 | 1 | 0 | 0 |
| 0.4 | 0.072 | 1 | 1 | 0 | 0 |
| 0.4 | 0.074 | 1 | 1 | 0 | 0 |
| 0.4 | 0.076 | 1 | 1 | 0 | 0 |
| 0.4 | 0.078 | 1 | 1 | 0 | 0 |
| 0.4 | 0.08 | 1 | 1 | 0 | 0 |
| 0.4 | 0.082 | 1 | 1 | 0 | 0 |
| 0.4 | 0.084 | 1 | 1 | 0 | 0 |
| 0.4 | 0.086 | 1 | 1 | 0 | 0 |
| 0.4 | 0.088 | 1 | 1 | 0 | 0 |
| 0.4 | 0.09 | 1 | 1 | 0 | 0 |
| 0.4 | 0.092 | 1 | 1 | 0 | 0 |
| 0.4 | 0.094 | 1 | 1 | 0 | 0 |
| 0.4 | 0.096 | 1 | 1 | 0 | 0 |
| 0.4 | 0.098 | 1 | 1 | 0 | 0 |
| 0.4 | 0.1 | 1 | 1 | 0 | 0 |
| 0.5 | 0.002 | 1 | 1 | 0 | 0 |
| 0.5 | 0.004 | 1 | 1 | 0 | 0 |
| 0.5 | 0.006 | 1 | 1 | 0 | 0 |
| 0.5 | 0.008 | 1 | 1 | 0 | 0 |
| 0.5 | 0.01 | 1 | 1 | 0 | 0 |
| 0.5 | 0.012 | 1 | 1 | 0 | 0 |
| 0.5 | 0.014 | 1 | 1 | 0 | 0 |
| 0.5 | 0.016 | 1 | 1 | 0 | 0 |
| 0.5 | 0.018 | 1 | 1 | 0 | 0 |
| 0.5 | 0.02 | 1 | 1 | 0 | 0 |
| 0.5 | 0.022 | 1 | 1 | 0 | 0 |
| 0.5 | 0.024 | 1 | 1 | 0 | 0 |
| 0.5 | 0.026 | 1 | 1 | 0 | 0 |
| 0.5 | 0.028 | 1 | 1 | 0 | 0 |
| 0.5 | 0.03 | 1 | 1 | 0 | 0 |
| 0.5 | 0.032 | 1 | 1 | 0 | 0 |
| 0.5 | 0.034 | 1 | 1 | 0 | 0 |
| 0.5 | 0.036 | 1 | 1 | 0 | 0 |
| 0.5 | 0.038 | 1 | 1 | 0 | 0 |
| 0.5 | 0.04 | 1 | 1 | 0 | 0 |
| 0.5 | 0.042 | 1 | 1 | 0 | 0 |
| 0.5 | 0.044 | 1 | 1 | 0 | 0 |
| 0.5 | 0.046 | 1 | 1 | 0 | 0 |
| 0.5 | 0.048 | 1 | 1 | 0 | 0 |
| 0.5 | 0.05 | 1 | 1 | 0 | 0 |
| 0.5 | 0.052 | 1 | 1 | 0 | 0 |
| 0.5 | 0.054 | 1 | 1 | 0 | 0 |
| 0.5 | 0.056 | 1 | 1 | 0 | 0 |
| 0.5 | 0.058 | 1 | 1 | 0 | 0 |
| 0.5 | 0.06 | 1 | 1 | 0 | 0 |
| 0.5 | 0.062 | 1 | 1 | 0 | 0 |
| 0.5 | 0.064 | 1 | 1 | 0 | 0 |
| 0.5 | 0.066 | 1 | 1 | 0 | 0 |
| 0.5 | 0.068 | 1 | 1 | 0 | 0 |
| 0.5 | 0.07 | 1 | 1 | 0 | 0 |
| 0.5 | 0.072 | 1 | 1 | 0 | 0 |
| 0.5 | 0.074 | 1 | 1 | 0 | 0 |
| 0.5 | 0.076 | 1 | 1 | 0 | 0 |
| 0.5 | 0.078 | 1 | 1 | 0 | 0 |
| 0.5 | 0.08 | 1 | 1 | 0 | 0 |
| 0.5 | 0.082 | 1 | 1 | 0 | 0 |
| 0.5 | 0.084 | 1 | 1 | 0 | 0 |
| 0.5 | 0.086 | 1 | 1 | 0 | 0 |
| 0.5 | 0.088 | 1 | 1 | 0 | 0 |
| 0.5 | 0.09 | 1 | 1 | 0 | 0 |
| 0.5 | 0.092 | 1 | 1 | 0 | 0 |
| 0.5 | 0.094 | 1 | 1 | 0 | 0 |
| 0.5 | 0.096 | 1 | 1 | 0 | 0 |
| 0.5 | 0.098 | 1 | 1 | 0 | 0 |
| 0.5 | 0.1 | 1 | 1 | 0 | 0 |
| 0.6 | 0.002 | 1 | 1 | 0 | 0 |
| 0.6 | 0.004 | 1 | 1 | 0 | 0 |
| 0.6 | 0.006 | 1 | 1 | 0 | 0 |
| 0.6 | 0.008 | 1 | 1 | 0 | 0 |
| 0.6 | 0.01 | 1 | 1 | 0 | 0 |
| 0.6 | 0.012 | 1 | 1 | 0 | 0 |
| 0.6 | 0.014 | 1 | 1 | 0 | 0 |
| 0.6 | 0.016 | 1 | 1 | 0 | 0 |
| 0.6 | 0.018 | 1 | 1 | 0 | 0 |
| 0.6 | 0.02 | 1 | 1 | 0 | 0 |
| 0.6 | 0.022 | 1 | 1 | 0 | 0 |
| 0.6 | 0.024 | 1 | 1 | 0 | 0 |
| 0.6 | 0.026 | 1 | 1 | 0 | 0 |
| 0.6 | 0.028 | 1 | 1 | 0 | 0 |
| 0.6 | 0.03 | 1 | 1 | 0 | 0 |
| 0.6 | 0.032 | 1 | 1 | 0 | 0 |
| 0.6 | 0.034 | 1 | 1 | 0 | 0 |
| 0.6 | 0.036 | 1 | 1 | 0 | 0 |
| 0.6 | 0.038 | 1 | 1 | 0 | 0 |
| 0.6 | 0.04 | 1 | 1 | 0 | 0 |
| 0.6 | 0.042 | 1 | 1 | 0 | 0 |
| 0.6 | 0.044 | 1 | 1 | 0 | 0 |
| 0.6 | 0.046 | 1 | 1 | 0 | 0 |
| 0.6 | 0.048 | 1 | 1 | 0 | 0 |
| 0.6 | 0.05 | 1 | 1 | 0 | 0 |
| 0.6 | 0.052 | 1 | 1 | 0 | 0 |
| 0.6 | 0.054 | 1 | 1 | 0 | 0 |
| 0.6 | 0.056 | 1 | 1 | 0 | 0 |
| 0.6 | 0.058 | 1 | 1 | 0 | 0 |
| 0.6 | 0.06 | 1 | 1 | 0 | 0 |
| 0.6 | 0.062 | 1 | 1 | 0 | 0 |
| 0.6 | 0.064 | 1 | 1 | 0 | 0 |
| 0.6 | 0.066 | 1 | 1 | 0 | 0 |
| 0.6 | 0.068 | 1 | 1 | 0 | 0 |
| 0.6 | 0.07 | 1 | 1 | 0 | 0 |
| 0.6 | 0.072 | 1 | 1 | 0 | 0 |
| 0.6 | 0.074 | 1 | 1 | 0 | 0 |
| 0.6 | 0.076 | 1 | 1 | 0 | 0 |
| 0.6 | 0.078 | 1 | 1 | 0 | 0 |
| 0.6 | 0.08 | 1 | 1 | 0 | 0 |
| 0.6 | 0.082 | 1 | 1 | 0 | 0 |
| 0.6 | 0.084 | 1 | 1 | 0 | 0 |
| 0.6 | 0.086 | 1 | 1 | 0 | 0 |
| 0.6 | 0.088 | 1 | 1 | 0 | 0 |
| 0.6 | 0.09 | 1 | 1 | 0 | 0 |
| 0.6 | 0.092 | 1 | 1 | 0 | 0 |
| 0.6 | 0.094 | 1 | 1 | 0 | 0 |
| 0.6 | 0.096 | 1 | 1 | 0 | 0 |
| 0.6 | 0.098 | 1 | 1 | 0 | 0 |
| 0.6 | 0.1 | 1 | 1 | 0 | 0 |
| 0.7 | 0.002 | 1 | 1 | 0 | 0 |
| 0.7 | 0.004 | 1 | 1 | 0 | 0 |
| 0.7 | 0.006 | 1 | 1 | 0 | 0 |
| 0.7 | 0.008 | 1 | 1 | 0 | 0 |
| 0.7 | 0.01 | 1 | 1 | 0 | 0 |
| 0.7 | 0.012 | 1 | 1 | 0 | 0 |
| 0.7 | 0.014 | 1 | 1 | 0 | 0 |
| 0.7 | 0.016 | 1 | 1 | 0 | 0 |
| 0.7 | 0.018 | 1 | 1 | 0 | 0 |
| 0.7 | 0.02 | 1 | 1 | 0 | 0 |
| 0.7 | 0.022 | 1 | 1 | 0 | 0 |
| 0.7 | 0.024 | 1 | 1 | 0 | 0 |
| 0.7 | 0.026 | 1 | 1 | 0 | 0 |
| 0.7 | 0.028 | 1 | 1 | 0 | 0 |
| 0.7 | 0.03 | 1 | 1 | 0 | 0 |
| 0.7 | 0.032 | 1 | 1 | 0 | 0 |
| 0.7 | 0.034 | 1 | 1 | 0 | 0 |
| 0.7 | 0.036 | 1 | 1 | 0 | 0 |
| 0.7 | 0.038 | 1 | 1 | 0 | 0 |
| 0.7 | 0.04 | 1 | 1 | 0 | 0 |
| 0.7 | 0.042 | 1 | 1 | 0 | 0 |
| 0.7 | 0.044 | 1 | 1 | 0 | 0 |
| 0.7 | 0.046 | 1 | 1 | 0 | 0 |
| 0.7 | 0.048 | 1 | 1 | 0 | 0 |
| 0.7 | 0.05 | 1 | 1 | 0 | 0 |
| 0.7 | 0.052 | 1 | 1 | 0 | 0 |
| 0.7 | 0.054 | 1 | 1 | 0 | 0 |
| 0.7 | 0.056 | 1 | 1 | 0 | 0 |
| 0.7 | 0.058 | 1 | 1 | 0 | 0 |
| 0.7 | 0.06 | 1 | 1 | 0 | 0 |
| 0.7 | 0.062 | 1 | 1 | 0 | 0 |
| 0.7 | 0.064 | 1 | 1 | 0 | 0 |
| 0.7 | 0.066 | 1 | 1 | 0 | 0 |
| 0.7 | 0.068 | 1 | 1 | 0 | 0 |
| 0.7 | 0.07 | 1 | 1 | 0 | 0 |
| 0.7 | 0.072 | 1 | 1 | 0 | 0 |
| 0.7 | 0.074 | 1 | 1 | 0 | 0 |
| 0.7 | 0.076 | 1 | 1 | 0 | 0 |
| 0.7 | 0.078 | 1 | 1 | 0 | 0 |
| 0.7 | 0.08 | 1 | 1 | 0 | 0 |
| 0.7 | 0.082 | 1 | 1 | 0 | 0 |
| 0.7 | 0.084 | 1 | 1 | 0 | 0 |
| 0.7 | 0.086 | 1 | 1 | 0 | 0 |
| 0.7 | 0.088 | 1 | 1 | 0 | 0 |
| 0.7 | 0.09 | 1 | 1 | 0 | 0 |
| 0.7 | 0.092 | 1 | 1 | 0 | 0 |
| 0.7 | 0.094 | 1 | 1 | 0 | 0 |
| 0.7 | 0.096 | 1 | 1 | 0 | 0 |
| 0.7 | 0.098 | 1 | 1 | 0 | 0 |
| 0.7 | 0.1 | 1 | 1 | 0 | 0 |
| 0.8 | 0.002 | 1 | 1 | 0 | 0 |
| 0.8 | 0.004 | 1 | 1 | 0 | 0 |
| 0.8 | 0.006 | 1 | 1 | 0 | 0 |
| 0.8 | 0.008 | 1 | 1 | 0 | 0 |
| 0.8 | 0.01 | 1 | 1 | 0 | 0 |
| 0.8 | 0.012 | 1 | 1 | 0 | 0 |
| 0.8 | 0.014 | 1 | 1 | 0 | 0 |
| 0.8 | 0.016 | 1 | 1 | 0 | 0 |
| 0.8 | 0.018 | 1 | 1 | 0 | 0 |
| 0.8 | 0.02 | 1 | 1 | 0 | 0 |
| 0.8 | 0.022 | 1 | 1 | 0 | 0 |
| 0.8 | 0.024 | 1 | 1 | 0 | 0 |
| 0.8 | 0.026 | 1 | 1 | 0 | 0 |
| 0.8 | 0.028 | 1 | 1 | 0 | 0 |
| 0.8 | 0.03 | 1 | 1 | 0 | 0 |
| 0.8 | 0.032 | 1 | 1 | 0 | 0 |
| 0.8 | 0.034 | 1 | 1 | 0 | 0 |
| 0.8 | 0.036 | 1 | 1 | 0 | 0 |
| 0.8 | 0.038 | 1 | 1 | 0 | 0 |
| 0.8 | 0.04 | 1 | 1 | 0 | 0 |
| 0.8 | 0.042 | 1 | 1 | 0 | 0 |
| 0.8 | 0.044 | 1 | 1 | 0 | 0 |
| 0.8 | 0.046 | 1 | 1 | 0 | 0 |
| 0.8 | 0.048 | 1 | 1 | 0 | 0 |
| 0.8 | 0.05 | 1 | 1 | 0 | 0 |
| 0.8 | 0.052 | 1 | 1 | 0 | 0 |
| 0.8 | 0.054 | 1 | 1 | 0 | 0 |
| 0.8 | 0.056 | 1 | 1 | 0 | 0 |
| 0.8 | 0.058 | 1 | 1 | 0 | 0 |
| 0.8 | 0.06 | 1 | 1 | 0 | 0 |
| 0.8 | 0.062 | 1 | 1 | 0 | 0 |
| 0.8 | 0.064 | 1 | 1 | 0 | 0 |
| 0.8 | 0.066 | 1 | 1 | 0 | 0 |
| 0.8 | 0.068 | 1 | 1 | 0 | 0 |
| 0.8 | 0.07 | 1 | 1 | 0 | 0 |
| 0.8 | 0.072 | 1 | 1 | 0 | 0 |
| 0.8 | 0.074 | 1 | 1 | 0 | 0 |
| 0.8 | 0.076 | 1 | 1 | 0 | 0 |
| 0.8 | 0.078 | 1 | 1 | 0 | 0 |
| 0.8 | 0.08 | 1 | 1 | 0 | 0 |
| 0.8 | 0.082 | 1 | 1 | 0 | 0 |
| 0.8 | 0.084 | 1 | 1 | 0 | 0 |
| 0.8 | 0.086 | 1 | 1 | 0 | 0 |
| 0.8 | 0.088 | 1 | 1 | 0 | 0 |
| 0.8 | 0.09 | 1 | 1 | 0 | 0 |
| 0.8 | 0.092 | 1 | 1 | 0 | 0 |
| 0.8 | 0.094 | 1 | 1 | 0 | 0 |
| 0.8 | 0.096 | 1 | 1 | 0 | 0 |
| 0.8 | 0.098 | 1 | 1 | 0 | 0 |
| 0.8 | 0.1 | 1 | 1 | 0 | 0 |
| 0.9 | 0.002 | 1 | 1 | 0 | 0 |
| 0.9 | 0.004 | 1 | 1 | 0 | 0 |
| 0.9 | 0.006 | 1 | 1 | 0 | 0 |
| 0.9 | 0.008 | 1 | 1 | 0 | 0 |
| 0.9 | 0.01 | 1 | 1 | 0 | 0 |
| 0.9 | 0.012 | 1 | 1 | 0 | 0 |
| 0.9 | 0.014 | 1 | 1 | 0 | 0 |
| 0.9 | 0.016 | 1 | 1 | 0 | 0 |
| 0.9 | 0.018 | 1 | 1 | 0 | 0 |
| 0.9 | 0.02 | 1 | 1 | 0 | 0 |
| 0.9 | 0.022 | 1 | 1 | 0 | 0 |
| 0.9 | 0.024 | 1 | 1 | 0 | 0 |
| 0.9 | 0.026 | 1 | 1 | 0 | 0 |
| 0.9 | 0.028 | 1 | 1 | 0 | 0 |
| 0.9 | 0.03 | 1 | 1 | 0 | 0 |
| 0.9 | 0.032 | 1 | 1 | 0 | 0 |
| 0.9 | 0.034 | 1 | 1 | 0 | 0 |
| 0.9 | 0.036 | 1 | 1 | 0 | 0 |
| 0.9 | 0.038 | 1 | 1 | 0 | 0 |
| 0.9 | 0.04 | 1 | 1 | 0 | 0 |
| 0.9 | 0.042 | 1 | 1 | 0 | 0 |
| 0.9 | 0.044 | 1 | 1 | 0 | 0 |
| 0.9 | 0.046 | 1 | 1 | 0 | 0 |
| 0.9 | 0.048 | 1 | 1 | 0 | 0 |
| 0.9 | 0.05 | 1 | 1 | 0 | 0 |
| 0.9 | 0.052 | 1 | 1 | 0 | 0 |
| 0.9 | 0.054 | 1 | 1 | 0 | 0 |
| 0.9 | 0.056 | 1 | 1 | 0 | 0 |
| 0.9 | 0.058 | 1 | 1 | 0 | 0 |
| 0.9 | 0.06 | 1 | 1 | 0 | 0 |
| 0.9 | 0.062 | 1 | 1 | 0 | 0 |
| 0.9 | 0.064 | 1 | 1 | 0 | 0 |
| 0.9 | 0.066 | 1 | 1 | 0 | 0 |
| 0.9 | 0.068 | 1 | 1 | 0 | 0 |
| 0.9 | 0.07 | 1 | 1 | 0 | 0 |
| 0.9 | 0.072 | 1 | 1 | 0 | 0 |
| 0.9 | 0.074 | 1 | 1 | 0 | 0 |
| 0.9 | 0.076 | 1 | 1 | 0 | 0 |
| 0.9 | 0.078 | 1 | 1 | 0 | 0 |
| 0.9 | 0.08 | 1 | 1 | 0 | 0 |
| 0.9 | 0.082 | 1 | 1 | 0 | 0 |
| 0.9 | 0.084 | 1 | 1 | 0 | 0 |
| 0.9 | 0.086 | 1 | 1 | 0 | 0 |
| 0.9 | 0.088 | 1 | 1 | 0 | 0 |
| 0.9 | 0.09 | 1 | 1 | 0 | 0 |
| 0.9 | 0.092 | 1 | 1 | 0 | 0 |
| 0.9 | 0.094 | 1 | 1 | 0 | 0 |
| 0.9 | 0.096 | 1 | 1 | 0 | 0 |
| 0.9 | 0.098 | 1 | 1 | 0 | 0 |
| 0.9 | 0.1 | 1 | 1 | 0 | 0 |
| 1 | 0.002 | 1 | 1 | 0 | 0 |
| 1 | 0.004 | 1 | 1 | 0 | 0 |
| 1 | 0.006 | 1 | 1 | 0 | 0 |
| 1 | 0.008 | 1 | 1 | 0 | 0 |
| 1 | 0.01 | 1 | 1 | 0 | 0 |
| 1 | 0.012 | 1 | 1 | 0 | 0 |
| 1 | 0.014 | 1 | 1 | 0 | 0 |
| 1 | 0.016 | 1 | 1 | 0 | 0 |
| 1 | 0.018 | 1 | 1 | 0 | 0 |
| 1 | 0.02 | 1 | 1 | 0 | 0 |
| 1 | 0.022 | 1 | 1 | 0 | 0 |
| 1 | 0.024 | 1 | 1 | 0 | 0 |
| 1 | 0.026 | 1 | 1 | 0 | 0 |
| 1 | 0.028 | 1 | 1 | 0 | 0 |
| 1 | 0.03 | 1 | 1 | 0 | 0 |
| 1 | 0.032 | 1 | 1 | 0 | 0 |
| 1 | 0.034 | 1 | 1 | 0 | 0 |
| 1 | 0.036 | 1 | 1 | 0 | 0 |
| 1 | 0.038 | 1 | 1 | 0 | 0 |
| 1 | 0.04 | 1 | 1 | 0 | 0 |
| 1 | 0.042 | 1 | 1 | 0 | 0 |
| 1 | 0.044 | 1 | 1 | 0 | 0 |
| 1 | 0.046 | 1 | 1 | 0 | 0 |
| 1 | 0.048 | 1 | 1 | 0 | 0 |
| 1 | 0.05 | 1 | 1 | 0 | 0 |
| 1 | 0.052 | 1 | 1 | 0 | 0 |
| 1 | 0.054 | 1 | 1 | 0 | 0 |
| 1 | 0.056 | 1 | 1 | 0 | 0 |
| 1 | 0.058 | 1 | 1 | 0 | 0 |
| 1 | 0.06 | 1 | 1 | 0 | 0 |
| 1 | 0.062 | 1 | 1 | 0 | 0 |
| 1 | 0.064 | 1 | 1 | 0 | 0 |
| 1 | 0.066 | 1 | 1 | 0 | 0 |
| 1 | 0.068 | 1 | 1 | 0 | 0 |
| 1 | 0.07 | 1 | 1 | 0 | 0 |
| 1 | 0.072 | 1 | 1 | 0 | 0 |
| 1 | 0.074 | 1 | 1 | 0 | 0 |
| 1 | 0.076 | 1 | 1 | 0 | 0 |
| 1 | 0.078 | 1 | 1 | 0 | 0 |
| 1 | 0.08 | 1 | 1 | 0 | 0 |
| 1 | 0.082 | 1 | 1 | 0 | 0 |
| 1 | 0.084 | 1 | 1 | 0 | 0 |
| 1 | 0.086 | 1 | 1 | 0 | 0 |
| 1 | 0.088 | 1 | 1 | 0 | 0 |
| 1 | 0.09 | 1 | 1 | 0 | 0 |
| 1 | 0.092 | 1 | 1 | 0 | 0 |
| 1 | 0.094 | 1 | 1 | 0 | 0 |
| 1 | 0.096 | 1 | 1 | 0 | 0 |
| 1 | 0.098 | 1 | 1 | 0 | 0 |
| 1 | 0.1 | 1 | 1 | 0 | 0 |

**Table S3. Implicated metabolites based on multiple modeling approaches in autistic males**

| Metabolite | Class. I | Class. II | VIP | p-value | Fold Change | Approaches Implicated |
| --- | --- | --- | --- | --- | --- | --- |
| Lys Lys Thr | -- | -- | 4.526 | <0.001 | <0.05 | LR,RF,SVM |
| Phe Phe Thr | -- | -- | 4.582 | 0.003 | <0.05 | LR,RF,SVM |
| Prostaglandin E2 ethanolamide | Lipids and lipid-like molecules | Fatty Acyls | 4.513 | 0.014 | <0.05 | LR,RF,SVM |
| 5-Nonadecylresorcinol | Benzenoids | Phenols | 4.610 | <0.001 | <0.05 | LR,RF,SVM |
| 21-Acetoxy-11beta, 17-dihydroxy-6alpha-methylpregn-4-ene-3,20-dione;17-Hydroxy-6alpha-methylcorticosterone 21-acetate | Lipids and lipid-like molecules | Steroids and steroid derivatives | 3.362 | 0.062 | <0.05 | LR,RF,SVM |
| Karacoline | Lipids and lipid-like molecules | Prenol lipids | 4.510 | 0.002 | <0.05 | LR,RF,SVM, XGB |
| Icaceine | Lipids and lipid-like molecules | Steroids and steroid derivatives | 4.553 | <0.001 | <0.05 | LR,RF,SVM |
| Psoromic acid | Phenylpropanoids and polyketides | Depsides and depsidones | 1.540 | 0.090 | <0.05 | LR,RF,SVM |
| Phosphonic acid, P-[[4-[(1-oxotetradecyl)amino]phenyl]methyl]- | Benzenoids | Benzene and substituted derivatives | 4.515 | 0.009 | <0.05 | LR,RF,SVM, ,XGB |

VIP: variable importance in projection; Lys Lys Thr: Lysine Lysine Threonine; Phe Phe Thr: Phenylalanine Phenylalanine Threonine: LR: logistic regression; RF: random forest; SVM: support vector machine classification; XGB: extreme gradient boosting


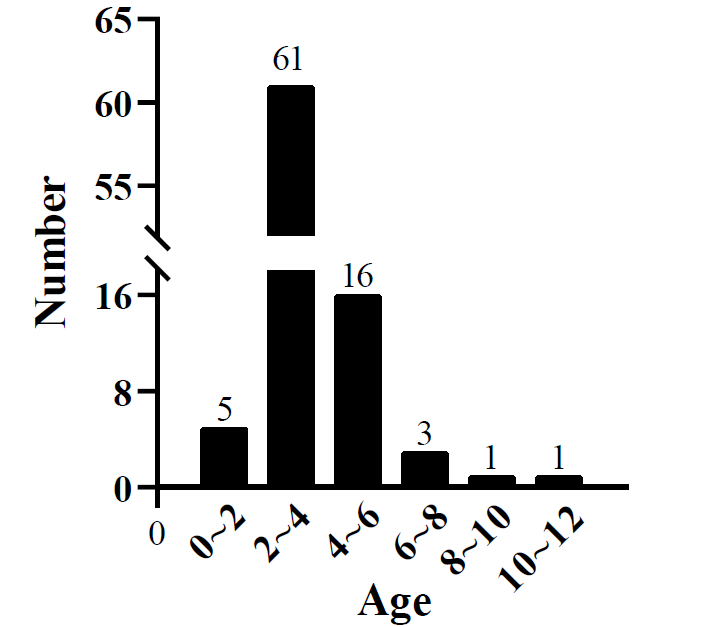


**Figure S1. The age distribution of all participants**


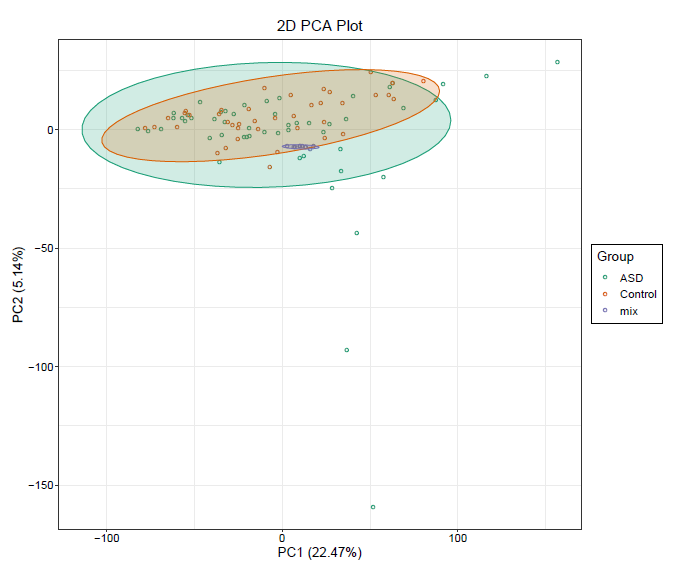


**Figure S2. PCA map of autistic children samples, typically developing samples and QC samples.**

PCA: principal component analysis; QC: quality control
